# Evaluation of Human and Viral Methylation, in Addition to Partial Genotyping, for a Molecular Triage Strategy in Women Under Active Surveillance for CIN2

**DOI:** 10.3390/cancers18132067

**Published:** 2026-06-25

**Authors:** Silvia Gori, Helena Frayle, Alessio Pagan, Marika Soldà, Cesare Romagnolo, Egle Insacco, Licia Laurino, Mario Matteucci, Giuseppe Sordi, Enrico Busato, Manuel Zorzi, Tiziano Maggino, Annarosa Del Mistro

**Affiliations:** 1 Immunology and Diagnostic Molecular Oncology Unit, Veneto Institute of Oncology IOV-IRCCS, Via Gattamelata 64, 35128 Padova, Italyannarosa.delmistro@iov.veneto.it (A.D.M.); 2 Ospedale Ca’ Foncello, Local Health Unit Marca Trevigiana, Piazzale dell’Ospedale 1, 31100 Treviso, Italy; 3 Obstetrics and Gynecology, Ospedale Città del Piave, Via Nazario Sauro 25, 30027 San Donà di Piave, Italy; 4 Ospedale dell’Angelo, Local Health Unit Serenissima, Via Don Tosatto 147, 30174 Venezia, Italy; 5 Obstetrics and Gynecology, Azienda Ospedale Università, Via Giustiniani 2, 35128 Padova, Italy; 6 Ospedale San Bonifacio, Local Health Unit Scaligera, Via Circonvallazione 1, 37047 Verona, Italy; 7 Veneto Tumour Registry, Azienda Zero, Via Jacopo Avanzo 35, 35132 Padova, Italy

**Keywords:** DNA methylation, papillomavirus infections, genotype, biomarkers, cervical cancer screening

## Abstract

**Background/Objective**: Cervical intraepithelial neoplasia grade 2 (CIN2) shows heterogeneous clinical behavior, with substantial rates of spontaneous regression under active surveillance. Reliable molecular biomarkers are needed to distinguish regressive from transforming lesions and reduce overtreatment. We evaluated the prognostic role of host and viral DNA methylation, alone and combined with HPV genotyping, in predicting CIN2 regression. **Methods**: This subanalysis derives from a prospective, multicenter Italian cohort of women with histologically confirmed CIN2 managed conservatively. Among 319 enrolled women, 134 with single HPV infections and valid host (FAM19A4/miR124-2) and viral (HPV L1 region) methylation results were included. HPV genotyping was performed with partial stratification (HPV16/18 vs. non-16/18). Clinical outcomes at 24 months were classified as regression versus persistence/progression. Logistic regression models assessed associations between biomarkers and regression. **Results**: At 24 months, 50% of women showed regression. Host and viral methylation positivity rates were more frequent in non-regressive lesions (40.3% vs. 19.4%, *p* = 0.01, and 52.2% vs. 32.8%, *p* = 0.02, respectively). Negative host methylation was significantly associated with regression (Odds Ratio OR = 0.37, 95% CI 0.17–0.81, *p* = 0.02), as was negative viral methylation (OR = 0.47, 95% CI 0.23–0.96, *p* = 0.04). **Conclusions**: Both host and viral methylation are inversely associated with CIN2 regression. Combining methylation markers did not substantially improve predictive accuracy; however, methylation negativity emerged as a potential molecular reassurance marker. When integrated with HPV genotyping, the highest probability of regression was observed among women with non-HPV16/18 infections and negative methylation results. These results endorse DNA methylation testing as a molecular tool for the conservative management of CIN2.

## Simple Summary

Cervical precancerous lesions classified as grade 2 show variable behavior: some progress, while many regress spontaneously. Current clinical management often leads to unnecessary treatments, which may affect future pregnancies. Therefore, better tools are needed to identify women at low risk who can be safely monitored without immediate intervention. In this study, we evaluated changes in DNA, called methylation, in both human cells and the virus responsible for cervical cancer, to predict whether lesions are likely to regress. We found that the absence of these molecular changes is strongly associated with lesion regression. These findings suggest that methylation testing could help guide more personalized management, reduce overtreatment, and improve patient care. This approach may support the development of more precise and less invasive strategies in cervical cancer prevention.

## 1. Introduction

Cervical intraepithelial neoplasia grade 2 (CIN2) represents a biologically heterogeneous lesion within the cervical carcinogenesis continuum. Although it carries a recognized risk of progression to CIN3 or invasive carcinoma, a substantial proportion of CIN2 lesions—estimated between 40% and 60%—regress spontaneously, particularly among younger women [1,2]. This variability in clinical behavior has driven increasing interest in conservative management strategies, particularly for women wishing to preserve fertility.

### 1.1. Conservative Management of CIN2

Historically, excisional treatment has been the standard for all histologic CIN2+ lesions. However, evidence has shown that routine excision results in significant overtreatment, exposing women to potential obstetric complications such as preterm delivery and low birth weight [3,4]. Consequently, major international guidelines—including ASCCP (2020) and European recommendations [5]—support active surveillance for selected women, particularly those under 30 years of age or with reproductive desire, provided that colposcopy is satisfactory and compliance with follow-up is ensured [6]. Active surveillance typically involves cytology, HPV testing, and colposcopy at defined intervals, with excision reserved for lesions that persist or progress.

### 1.2. Host DNA Methylation Biomarkers

Aberrant methylation of host-cell genes is a key epigenetic hallmark of cervical carcinogenesis. Methylation of tumor suppressor and regulatory genes, including FAM19A4, miR124-2, CADM1, and MAL, progressively increases from CIN1 through CIN3 to invasive cancer [7,8]. The FAM19A4/miR124-2 methylation panel has shown excellent performance as a triage test for HPV-positive women, with high specificity and reproducibility across sample types and screening settings [9].

Importantly, studies have demonstrated that methylation status provides clinically relevant information in the conservative management of CIN2. Methylation-positive CIN2 lesions are less likely to regress and often display transforming infection markers (p16/Ki-67 positivity and absence of HPV E4 expression), whereas methylation-negative lesions have higher regression rates [10,11]. In a large Dutch cohort, methylation-negative CIN2 lesions regressed in over 70% of cases within 24 months of observation, supporting methylation as a predictor of safe surveillance [10]. These findings position methylation testing as a promising biomarker to guide individualized management decisions and reduce unnecessary excisions in low-risk women.

### 1.3. Viral Methylation Biomarkers

In parallel, methylation of high-risk HPV genomes, particularly in the L1 and L2 regions, reflects viral integration and transcriptional control. High methylation levels correlate with persistence and lesion severity [12,13,14]. HPV16 and HPV18 methylation levels increase with CIN grade and are associated with non-regressive, transforming infections [15,16]. Viral methylation thus represents an additional molecular dimension potentially complementing host methylation to refine risk stratification in conservatively managed CIN2.

### 1.4. Molecular Triage and Rationale for Combined Testing

In women diagnosed with CIN2 and managed conservatively, one of the main challenges is to identify robust and objective biomarkers capable of distinguishing truly regressive lesions from those likely to progress. Traditional markers such as HPV persistence, cytology, or p16/Ki-67 immunostaining provide only moderate predictive value [17,18]. As a result, there is growing interest in developing molecular triage strategies that can accurately determine which CIN2 lesions are likely to regress versus those at risk of persistence or progression, enabling a more personalized approach to management.

DNA methylation testing of both host and viral genes has emerged as one of the most promising molecular approaches in this setting. This makes methylation testing a strong candidate for a molecular triage tool to safely extend conservative management in appropriately selected women.

Whether the integration of host and viral methylation can improve risk stratification beyond HPV genotyping alone remains unclear. Viral methylation may reflect biological features of HPV persistence and transformation that are not fully captured by cytology or partial HPV genotyping. Therefore, the present study aimed to evaluate the comparative and combined predictive value of host and viral methylation markers, together with HPV genotyping, for identifying CIN2 lesions likely to regress during active surveillance. Combining both could potentially improve predictive accuracy by capturing distinct but complementary aspects of disease biology [19]. The addition of partial HPV genotyping (HPV16/18 vs. non-16/18 types) could further enhance discrimination, given the known higher oncogenic potential of HPV16 and HPV18 [20].

Despite these theoretical advantages, few studies have directly compared the predictive value of human and viral methylation within the same cohort of women under active CIN2 surveillance. The individual and combined contributions of these biomarkers, as well as their integration with HPV genotyping, remain to be fully clarified.

The present study is a sub-analysis of our prospective study, performed as part of the cervical cancer screening programs in the Veneto region and aimed at evaluating the clinical outcome of CIN2 lesions managed conservatively and the prognostic value of various biomarkers (HPV genotyping, p16/ki67 dual stain, and cellular methylation) tested in the cervical cells for predicting regression [21].

The purpose of this study is to explore the comparative and combined predictive performance of human and viral methylation markers—alone and in conjunction with HPV genotyping—in identifying CIN2 lesions likely to regress, thereby assessing their potential role as a molecularly informed triage strategy in the conservative management of CIN2.

## 2. Materials and Methods

### 2.1. Study Design and Population

This study is a subanalysis of a previously published prospective multicenter cohort study conducted in Italy, with the aim of identifying predictive biomarkers of regression in women with histologically confirmed cervical intraepithelial neoplasia grade 2 (CIN2) managed with active surveillance. Details of the main cohort design have been reported elsewhere [22]. Briefly, 319 women aged 25–45 years were enrolled between 2019 and 2021 across four participating centers. Eligibility criteria included full visualization of the transformation zone and lesion, no previous history of CIN2+ or cervical treatment, and absence of immunodeficiency or pregnancy at baseline. All participants were followed for 24 months, with repeat cytology, HPV testing, and colposcopy at 6-to-12-month intervals.

The present subanalysis was conducted on a subset of women for whom both host and viral methylation data were available at baseline.

The viral methylation protocol used in this study is restricted to the analysis of single HPV infections only. Therefore, from the original cohort of 319 women, 201 single HPV infections were identified and selected for potential inclusion. Among these, 14 cases were excluded due to missing follow-up outcome data. Of the remaining cases, 49 samples yielded invalid viral methylation results, leaving 138 valid viral methylation results. Finally, four additional cases were excluded due to invalid host methylation data, resulting in a total of 134 women included in the present analysis.

To assess the potential for selection bias, baseline demographic and clinical characteristics of women included in the present analysis were compared with those excluded because of multiple HPV infections, missing follow-up data, or invalid methylation results. Variables examined included age, baseline cytology, and outcomes.

Clinical outcomes at 24 months were classified as regression, persistence, or progression, based on histological and colposcopic follow-up findings, according to internationally accepted criteria.

### 2.2. Molecular Analyses

#### 2.2.1. HPV Genotyping

HPV genotyping was performed as previously described in the parent cohort study [22]. Briefly, cervical samples were initially tested using the Cobas 4800 HPV assay (Roche Diagnostics, Basel, Switzerland), which provides identification of HPV16 and HPV18 and pooled detection of other high-risk HPV types. Samples requiring further characterization were genotyped using validated PCR-based methods, with additional testing performed when necessary to assign a specific high-risk HPV genotype.

For the purposes of the present analysis, HPV types were categorized according to partial genotyping (HPV16/18 vs. non-HPV16/18). Extended genotype groupings were also considered in exploratory analyses, as described below.

HPV types were classified hierarchically and assigned to two (for partial genotyping: 16/18 and non-16/18) or three (for extended genotyping: 16/18, 31/33/35/45/52/58, and 39/51/56/59/66/68) groups, also in cases of multiple infections. Each woman was included in one group only.

#### 2.2.2. Host Methylation

Host-cell methylation was assessed using the QIAsure Methylation Test (Qiagen, Hilden, Germany), which evaluates methylation of the FAM19A4 and miR124-2 gene promoters. DNA extraction, bisulfite conversion, amplification, quality control procedures, and result interpretation were performed according to the manufacturer’s instructions and as previously reported [21,22].

Samples were classified as methylation-positive when at least one of the two markers was positive. Samples yielding invalid results after repeat testing were considered inadequate and excluded from the analysis.

#### 2.2.3. Viral Methylation

CpGs of interest for hrHPV methylation status were selected as described previously [23]. DNA modified with sodium bisulfite (EpiTect Bisulfite Kit, Qiagen) for complete conversion of unmethylated cytosine residues into uracil was used to evaluate the methylation status of the selected CpGs. Methylation analysis was performed by pyrosequencing after preliminary PCR reactions were set up with previously established primers for which validation in terms of consistency with type-specific PCR and reproducibility of methylation quantification was shown [23]. The newly generated consensus primers were used for L1-I. Two consensus primer couples were used for L1-I, classified as HPV16,31,33,35,52,58 and HPV39,45,51,59, respectively, whereas dedicated primers were needed for HPV56 and 18. Details on bisulfite conversion, primer sequences, PCR reaction profiles, and controls were given earlier in this document [23].

A summary of the CpG sites analyzed for each HPV genotype is provided in Supplementary Table S1.

Methylation assays by pyrosequencing were performed on a PyroMark Q96 instrument (Diatech Pharmacogenetics, Jesi, Italy). A “CpG mode” software Q24 version 2.0 was used, and this ensured comparable performance. Methylation at each CpG site was rendered by PyroMark for each sample as the proportion of methylated cytosines through the C/(C + T) ratio, reported as the percentage of methylation at a single CpG site and, when more than one CpG site was evaluated in one region (i.e., L1-I), as the mean methylation percentage of all the CpG sites in the studied gene region.

### 2.3. Statistical Analysis

Descriptive statistics were used to summarize biomarker distributions. Differences between regressive and non-regressive groups were assessed using the χ^2^ test or Fisher’s exact test for categorical variables and the Mann–Whitney U test for continuous variables.

To establish the optimal thresholds for positivity, Receiver Operating Characteristic (ROC) curve analysis was performed using CIN2 regression versus persistence/progression as the binary outcome.

Associations between biomarker status and CIN2 regression were evaluated using multivariable logistic regression analysis. Odds ratios (ORs) with 95% confidence intervals (CIs) were calculated. Separate models were run for (1) host methylation alone; (2) viral methylation alone; (3) the combination of both methylation markers (positive when at least one of the two is positive and negative only when both are negative); and (4) combined methylation plus partial HPV genotyping. Statistical significance was set at *p* < 0.05. All analyses were performed using R statistical software (version 4.4.2) [24].

## 3. Results

### 3.1. Study Population and Outcomes

A total of 134 women were included in this subanalysis, representing those from the original cohort with available results for both host and viral methylation testing. To evaluate the potential impact of the exclusions on study representativeness, women included in the viral methylation analysis (*n* = 134) were compared with those excluded from the original cohort (*n* = 185). No significant differences were observed in age, baseline cytology, or clinical outcome, suggesting that the analyzed sample was broadly representative of the original study population. The median age was 34 years (IQR = 29–38). At 24-month follow-up, 67 women (50%) demonstrated histological regression, while 67 (50%) had either persistent or progressive CIN2 lesions. Among non-regressive cases, 30 (44.8%) progressed to CIN3+, of which 2 were adenocarcinoma in situ, and 1 was invasive cancer (FIGO stage IA1). Their baseline characteristics, including cytology, HPV genotype, and methylation status, are reported in Supplementary Table S2. No significant differences in age, genotyping, or cytology were observed between the two outcome groups, regression vs. non-regression (*p* = 0.45, *p* = 0.34, and *p* = 0.47).

HPV16/18 infection was detected in 50% of the cohort and was more frequent in non-regressive lesions (58.2% vs. 41.8%, *p* = 0.05). The expression of p16/Ki67 was positive in 41.8% overall, also more prevalent among non-regressive cases (71.4% vs. 28.6%, *p* < 0.001) (Table 1).

### 3.2. Diagnostic Performance of Viral Methylation and ROC Analysis

**Figure 1:**
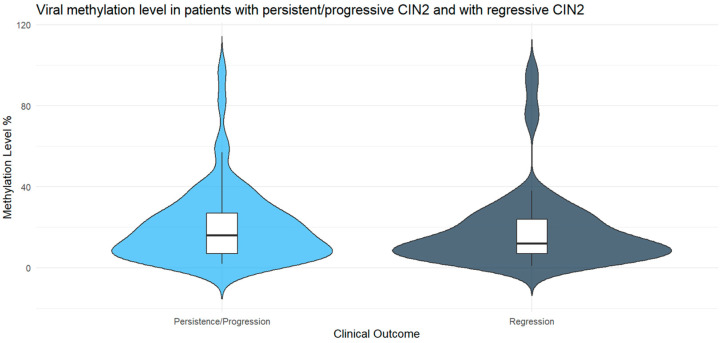
Violin plot. Distribution of viral DNA methylation levels according to clinical outcome (persistence/progression and regression) in women with conservatively managed CIN2.

Viral methylation levels were not significantly higher in non-regressive lesions (median = 16%) than in regressive ones (median = 12%, *p* = 0.38) (Figure 1).

To account for genotype-specific differences in viral methylation levels, HPV genotypes were grouped according to their methylation distributions observed in the study population. Pairwise comparisons showed that HPV18, HPV45, and HPV52 had significantly higher methylation levels than HPV16, whereas no significant differences were observed for HPV31, HPV33, HPV35, HPV39, HPV51, HPV56, HPV58, HPV59, HPV66, and HPV68 (Supplementary Table S3). Based on these findings, two groups were generated: (a) a group including HPV 16, 31, 33, 39, 51, 56, and 58 and (b) a group including HPV 18, 45, and 52.

Receiver Operating Characteristic (ROC) curve analysis was performed to determine the optimal viral methylation thresholds for predicting CIN2 regression (Youden Index).

Two cut-off values were identified: group (a) 15.5% (lower threshold, sensitivity = 49.2%, specificity = 69.5%, AUC = 0.57) and group (b) 30.5% (higher threshold, sensitivity = 100%, specificity = 63.6%, AUC = 0.73).

### 3.3. Distribution of Host and Viral Methylation According to Clinical Outcomes

The distribution of methylation markers differed significantly between women with regressive and non-regressive lesions.

Host-gene methylation (FAM19A4/miR124-2) was positive in 19.4% of regressive and 40.3% of non-regressive lesions (*p* = 0.01). Viral methylation was positive in 32.8% of regressive and 52.2% of non-regressive lesions (*p* = 0.02). No significant correlation was observed between host and viral methylation positivity (τ = 0.098, *p* > 0.26), suggesting they represent complementary molecular processes.

### 3.4. Association Between Methylation and CIN2 Regression

In univariate analysis, both human and viral methylation were inversely associated with CIN2 regression.

In multivariable logistic regression models, negative host methylation was significantly associated with lesion regression (OR = 0.37; 95% CI = 0.17–0.81; *p* = 0.02). Similarly, negative viral methylation predicted regression (OR = 0.47; 95% CI = 0.23–0.96; *p* = 0.04).

When both host and viral methylation markers were included jointly, a small increase in the model’s predictive accuracy was observed, suggesting that either biomarker alone provides sufficient discriminatory capacity for molecular triage purposes (Table 2).

### 3.5. Combined Model Including HPV Genotyping

When partial HPV genotyping was added to the model, negative methylation remained positively associated with regression, while partial genotyping was not significantly associated with regression.

When partial HPV genotyping was incorporated into the multivariable logistic regression models alongside either human or viral methylation status, the only significant association with lesion regression (as compared with HPV16/18 and positive methylation) was observed among women with non-HPV16/18 infections and negative methylation results. In contrast, no significant association was found when HPV16 or HPV18 infections were present, regardless of methylation status.

When a combined methylation variable—representing the cumulative (summed) status of human and viral methylation—was used in conjunction with partial HPV genotyping, an overall association with regression was maintained for methylation-negative results, irrespective of HPV genotype. In this model, methylation negativity was significantly associated with an increased likelihood of regression, suggesting that the absence of methylation marks, whether in human or viral targets, reflects a lower risk of lesion persistence or progression regardless of HPV type (Table 3).

## 4. Discussion

This subanalysis of a prospective cohort of women with conservatively managed CIN2 provides new insights into the prognostic role of host and viral DNA methylation as molecular biomarkers of lesion outcome. Our findings confirm that both host (FAM19A4/miR124-2) and viral methylation levels are inversely associated with regression, supporting their value in risk stratification during active surveillance. Moreover, the integration of methylation markers with partial HPV genotyping further refines the predictive framework, highlighting that the absence of methylation—either in human or viral targets—correlates with lesion regression, irrespective of HPV type.

### 4.1. Host and Viral Methylation as Predictors of Regression

Consistent with previous evidence [10,11,25], our study confirms that negative host methylation is significantly associated with spontaneous regression of CIN2. Similar findings have been reported by Kremer et al. in the CONCERVE study, where methylation-negative CIN2 lesions exhibited regression rates exceeding 70%, and by Vink et al., who demonstrated the clinical utility of FAM19A4/miR124-2 testing in younger HPV-positive women. Methylation positivity of FAM19A4 and/or miR124-2 probably reflects transcriptional silencing of tumor suppressor genes and a more advanced epigenetic state along the cervical neoplastic continuum. This biological signature appears to distinguish transforming infections, which are less likely to regress, from transient productive infections that can resolve spontaneously.

Similarly, viral methylation was associated with CIN2 persistence or progression, in line with previous reports showing that hypermethylation of HPV L1/L2 regions correlates with viral integration, reduced E2 transcriptional control, and increased oncogene expression [14,26,27]. An interesting finding was the marked variability in baseline methylation levels across HPV genotypes. In particular, HPV18, HPV45, and HPV52 exhibited higher methylation levels than HPV16 and most other high-risk types, requiring the use of distinct methylation thresholds. Although the biological basis of these differences remains incompletely understood, genotype-specific methylation patterns have previously been reported and may reflect differences in viral genome organization, integration dynamics, or epigenetic regulation. Nevertheless, the genotype groupings used in the present study were primarily derived from the observed methylation distributions and should therefore be considered exploratory until validated in independent cohorts. Our data indicate that lesions with low or absent viral methylation exhibit a higher probability of regression, suggesting that viral methylation provides complementary biological information to host methylation. The absence of correlation between the two markers in our cohort further supports the hypothesis that host and viral methylation capture distinct but convergent mechanisms of carcinogenic transformation—the former reflecting host epigenetic reprogramming and the latter viral genome integration and transcriptional deregulation.

However, the predictive performance of methylation markers was not absolute. A proportion of lesions that subsequently persisted or progressed remained methylation-negative at baseline, indicating that methylation status alone does not fully capture the biological heterogeneity of CIN2 lesions. Therefore, methylation results should be interpreted in conjunction with other clinical and virological factors.

### 4.2. Combined Methylation and Genotyping Models

When human and viral methylation were included simultaneously in multivariable models, the combined analysis did not substantially improve predictive accuracy over each marker alone. This suggests that, despite their complementary nature, both biomarkers may convey overlapping prognostic information with regard to the biological state of the lesion. Nevertheless, when partial HPV genotyping was added to the models, an interesting pattern emerged: regression was significantly associated with the combination of non-HPV16/18 infection and methylation negativity, whereas no such association was observed in HPV16/18-positive cases, irrespective of methylation status. This finding is biologically plausible. The intrinsically higher carcinogenic potential and persistence risk associated with HPV16 and HPV18 may attenuate the prognostic value of methylation negativity. This observation should be interpreted cautiously, as methylation negativity likely reflects a biologically less advanced lesion, irrespective of genotype. Larger studies are required to determine whether HPV16/18 infections truly modify the predictive significance of methylation status [28,29].

Importantly, when methylation was modeled as a cumulative variable combining both human and viral methylation results, methylation negativity remained strongly associated with regression, independently of HPV genotype. This suggests that the absence of methylation marks—regardless of their origin—reflects a biologically less advanced lesion and may represent a robust molecular indicator of regression potential. From a clinical perspective, these findings suggest that methylation negativity may contribute to risk stratification during conservative management. Nevertheless, the occurrence of progression among methylation-negative lesions indicates that methylation testing cannot currently be considered a stand-alone tool for clinical decision-making.

Methylation negativity may function as a molecular reassurance marker when interpreted together with established clinical, cytological, and virological parameters. However, further validation is required before this approach can be incorporated into routine clinical practice.

### 4.3. Clinical Implications for Conservative Management of CIN2

The identification of reliable molecular predictors of regression is critical to optimize the management of CIN2, reducing unnecessary excisional procedures without compromising oncologic safety. Our results add to a growing body of international evidence supporting the use of FAM19A4/miR124-2 methylation testing as a marker of transforming HPV infections and as a tool for identifying women suitable for conservative management [9,10,11,20,25]. The consistency of findings across different populations and study settings strengthens the rationale for incorporating methylation testing into active surveillance protocols, namely the ability of methylation negativity to identify lesions likely to regress, which supports its potential as a “molecular reassurance” marker to safely extend surveillance intervals or avoid overtreatment in selected cases.

Viral methylation, though technically more demanding, could complement host methylation testing, especially in HPV16/18-negative women, to further refine individualized management strategies. Nevertheless, the practical applicability of viral methylation testing remains limited by technical challenges. In the present study, a substantial proportion of samples yielded invalid viral methylation results, reducing the effective sample size and highlighting the need for further optimization and standardization of viral methylation assays before widespread clinical implementation. The development of combined methylation-based triage algorithms could ultimately enable a fully molecular follow-up approach, reducing repeated colposcopic assessment.

Although methylation negativity was associated with lesion regression, the present findings should not be interpreted as sufficient evidence to support immediate implementation of methylation-guided management strategies. Larger prospective studies are needed to establish the safety, reproducibility, and predictive performance of methylation-based algorithms before they can be incorporated into routine clinical care.

An important limitation should, however, be considered. This subanalysis included only women for whom viral methylation data were available (*n* = 134), which may limit its statistical power and introduce selection bias. Nevertheless, a comparison between included and excluded women showed no significant differences in age, baseline cytology, or clinical outcome, suggesting that the study population remained representative of the original cohort despite the reduction in sample size. The restricted sample size derives from the fact that the viral methylation assay applied in this study is validated only for single-type HPV infections, thus excluding multiple infections. This methodological constraint reduced the eligible cohort and led to the underrepresentation of women with multiple HPV infections. Such infections are frequently encountered in routine clinical practice and may differ biologically from single-type infections with respect to viral persistence, methylation patterns, and lesion evolution. Consequently, the molecular algorithm evaluated in this study cannot yet be directly generalized to the broader population of women undergoing CIN2 surveillance. Future studies should investigate whether similar methylation-based risk stratification strategies remain applicable in cohorts including multiple HPV infections. Furthermore, the use of partial genotyping (HPV16/18 vs. non-16/18) provides only limited stratification of viral risk, and further studies using extended genotyping panels are warranted.

## 5. Conclusions

In conclusion, the most clinically relevant finding of this study is the strong association between methylation negativity and spontaneous CIN2 regression. The absence of host and/or viral methylation marks was associated with an increased probability of spontaneous regression and may contribute to risk stratification during active surveillance. However, progression was also observed among methylation-negative lesions, indicating that methylation testing should currently be regarded as a promising investigational biomarker rather than a stand-alone clinical tool. The combination of methylation and HPV genotyping further refines this risk stratification, with the most favorable outcomes observed in women with non-HPV16/18 infections and negative methylation. Larger prospective studies integrating host and viral methylation with extended genotyping are warranted to confirm these findings and define clinically applicable algorithms for personalized management of CIN2. 

## Figures and Tables

**Table 1:** Baseline characteristics of the women in the CIN2 cohort finally included for the subanalysis (*n* = 134) by clinical outcome.

	Regression (*n* = 67)	Persistence/Progression (*n* = 67)	*p*-Value
**Age (years) *n* (%)**			
25–29	18 (26.9)	20 (29.9)	0.45
30–34	19 (28.4)	24 (35.8)	
35–39	14 (20.9)	14 (20.9)	
40–45	16 (23.9)	9 (13.4)	
**Genotyping *n* (%)**			
HPV16	25 (37.3)	37 (55.2)	0.34
HPV18	3 (4.5)	2 (3)	
HPV31	13 (19.4)	13 (19.4)	
HPV33	7 (10.4)	5 (7.5)	
HPV39	0 (0)	1 (1.5)	
HPV45	3 (4.5)	1 (1.5)	
HPV51	1 (1.5)	0 (0)	
HPV52	5 (7.5)	2 (3)	
HPV56	3 (4.5)	0 (0)	
HPV58	7 (10.4)	6 (9)	
**HPV partial genotyping *n* (%)**			
HPV16/18	28 (41.8)	39 (58.2)	0.058
HPV no16/18	39 (58.2)	28 (41.8)	
**Cytology *n* (%)**			
Negative	6 (9)	5 (7.5)	0.47
LSIL	25 (37.3)	22 (32.8)	
ASC-US	6 (9)	2 (3)	
HSIL	16 (23.9)	23 (34.3)	
ASC-H	9 (13.4)	10 (14.9)	
AGC	1 (1.5)	0 (0)	
Not available	4 (6)	5 (7.5)	
**p16/ki67 *n* (%)**			
Negative	43 (64.2)	25 (37.3)	<0.001
Positive	16 (23.9)	40 (59.7)	
Not available	8 (11.9)	2 (3)	

**Table 2:** Association between host and viral methylation status and clinical outcome (regression vs. persistence/progression) after 24 months of active surveillance.

Biomarker	OR	95% CI	*p*-Value
Host methylation			
Positive (reference)	-	-	-
Negative	0.37	0.17–0.81	0.02
Viral methylation			
Positive (reference)	-	-	-
Negative	0.47	0.23–0.96	0.04
Methylation (host + viral)			
Positive (reference)	-	-	-
Negative	0.30	0.14–0.61	0.001

**Table 3:** Multivariable logistic regression analysis evaluating the combined effect of host methylation, viral methylation, and partial HPV genotyping (HPV16/18 vs. non-16/18) on CIN2 regression at 24 months.

Biomarker	OR	95% CI	*p*-Value
Partial genotyping + Host methylation			
HPV16/18 + positive methylation (reference)	-	-	-
HPV no16/18 + positive methylation	0.80	0.20–3.30	0.80
HPV 16/18 + negative methylation	0.47	0.16–1.29	0.15
HPV no16/18 + negative methylation	0.25	0.09–0.66	0.01
Partial genotyping + Viral methylation			
HPV16/18 + positive methylation (reference)	-	-	-
HPV no16/18 + positive methylation	0.65	0.22–1.91	0.40
HPV 16/18 + negative methylation	0.55	0.20–1.50	0.30
HPV no16/18 + negative methylation	0.21	0.07–0.60	0.004
Partial genotyping + Methylation (host + viral)			
HPV16/18 + positive methylation (reference)	-	-	-
HPV no16/18 + positive methylation	0.64	0.25–1.64	0.40
HPV16/18 + negative methylation	0.35	0.12–0.98	0.05
HPV no16/18 + negative methylation	0.20	0.07–0.51	0.001

## Data Availability

The row data supporting the conclusion of this article will be made available by the authors upon request.

## Supplementary Materials

The following supporting information can be downloaded at: https://www.mdpi.com/article/10.3390/cancers18132067/s1, Table S1: Reference sequences for the twelve oncogenic HPV types and CpG positions; Table S2: Baseline characteristics of CIN3+ cases; Table S3: Pairwise comparisons of viral DNA methylation levels between individual high-risk HPV genotypes and HPV16.

## Abbreviations

The following abbreviations are used in this manuscript:

AGC	Atypical glandular cells
ASC-H	Atypical squamous cells cannot exclude high-grade
ASC-US	Atypical squamous cells of undetermined significance
ASCCP	American Society for Colposcopy and Cervical Pathology
AUC	Area under the curve
CI	Confidence interval
CIN	Cervical intraepithelial neoplasia
Ct	Cycle thresholds
FIGO	International Federation of Gynecology and Obstetrics
HPV	Human papillomavirus
hrHPV	High-risk human papillomavirus
HR HPV non16/18	HrHPV other than 16/18
HSIL	High-grade squamous intraepithelial lesion
LSIL	Low-grade squamous intraepithelial lesion
ODs	Odds ratio
ROC	Receiver operating characteristic
