# Supplementary material for: Evaluation of Human and Viral Methylation, in Addition to Partial Genotyping, for a Molecular Triage Strategy in Women Under Active Surveillance for CIN2
Source: Cancers (Basel). 2026 Jun 25;18(13):2067. doi: 10.3390/cancers18132067 (PMC13360136; doi:10.3390/cancers18132067)
Supplement: Supplementary file 1 [file cancers-18-02067-s001.zip › cancers-4318912-supplementary.pdf]

Table S1. Reference sequences for the twelve oncogenic HPV types and CpG positions.

HPV type	Reference Sequence	CpG position L1 I
16	NCBI: NC_001526.2	5601; 5606; 5609; 5616
18	GenBank: KC470224.1	5599; 5616
31	GenBank: J04353.1	5518; 5521; 5524; 5530
33	GenBank: M12732.1	5557; 5560; 5566; 5572
35	GenBank: M74117.1	5540; 5543; 5546; 5553
39	GenBank: KC470245.1	5682
45	GenBank: KC470260.1	5620; 5636
51	GenBank: GQ487711.1	13
52	GenBank: HQ537750.1	5613; 5616; 5622; 5628
56	GenBank: EF177179.1	5561; 5567; 5570; 5576
58	GenBank: GI222386	5606; 5609; 5615; 5621
59	GenBank: KC470266.1	5618

Table S2. Baseline characteristics of CIN3+ cases

Age	HPV	Cytology	Viral Methylation	Host Methylation	Histology
40	16	LSIL	20	POS/POS	Kinv
35	16	LSIL	11	NEG/NEG	AIS
32	31	LSIL	39	NEG/NEG	AIS

Table S3. Pairwise comparisons of viral DNA methylation levels between individual high-risk HPV genotypes and HPV16.

Comparison	Z	p-value
16-18	-3.189	0.001
16-31	0.964	0.335
16-33	-0.616	0.538
16-39	-1.021	0.307
16-45	-3.206	0.001
16-51	-1.481	0.139
16-52	-1.753	0.080
16-56	1.043	0.297
16-58	-0.226	0.821
